# Draft Genome Sequence of Bacillus wiedmannii Biovar *thuringiensis* ZZQ-138, Isolated from a Saline Lake

**DOI:** 10.1128/mra.00964-21

**Published:** 2022-02-10

**Authors:** Xiaojie Lan, Qi Wang, Tingjue Wu, Na Li, Hongxun Wang, Ziqiang Zheng

**Affiliations:** a College of Life Science and Technology, Wuhan Polytechnic University, Wuhan, China; Indiana University, Bloomington

## Abstract

Ecologically sound approaches to control mosquitoes like Anopheles stephensi, which are obligatory vectors for malaria transmission, are urgently needed because of increasing insecticide resistance. Bacteria from *Bacillus* are important resources. Here, we report the whole-genome sequence of Bacillus wiedmannii biovar *thuringiensis* ZZQ-138, which was isolated from sediment from a saline lake.

## ANNOUNCEMENT

Bacillus wiedmannii, an important member of the Bacillus cereus group, is a Gram-positive spore-forming bacterium that produces diverse pesticidal proteins (1). To isolate novel B. wiedmannii strains, we obtained samples from different ecological niches, such as a saline lake. Here, B. wiedmannii biovar *thuringiensis* ZZQ-138 was isolated with the classic spread plate method from sediment collected at a point that was seldom affected by tourists, at a depth of 3 m and a distance of 30 m from the shore of Caka Salt Lake, Qinghai province, China. The salt concentration of the sediment sample is about 6%.

ZZQ-138 was cultured in liquid salty (6%) Luria-Bertani (LB) medium at 28°C, and its genomic DNA at the logarithmic growth phase in the aforementioned medium was prepared with the previously modified alkaline lysis method of Andrup et al. (2). DNA library preparation was performed using the NEXTFLEX Rapid DNA-Seq kit after fragments 150 to 500 bp in size were generated with a Covaris M220 ultrasonicator, and the sequencing was conducted on an Illumina HiSeq X Ten sequencer.

A total of 13,211,952 paired-end reads of 150 bp were obtained, with a sequencing depth of >330-fold, after a quality check performed with fastp v0.20.0 (3) by default. We trimmed the raw data with the following steps: (i) remove the adapter sequences of the reads; (ii) shear the non-A, G, C, and T bases at the 5′ terminus of the reads; (iii) cut the terminus of the reads with low quality (Q scores of <20); (iv) remove the reads with >10% N; and (v) discard the reads shorter than 25 bp after adapter removal and quality trimming. The trimmed reads were assembled with the Assemble module of PGCGAP (4), which is a comprehensive, malleable, and easily installed prokaryotic genomic and comparative genomic analysis pipeline. The command line and parameters are as follows: pgcgap --Assemble --platform illumina --assembler auto --filter_length 200 --ReadsPath Reads/Illumina --reads1 _1.fastq.gz --reads2 _2.fastq.gz --kmmer 81 --threads 4 --suffix_len 11. Contigs of >200 bp were annotated using the NCBI Prokaryotic Genome Annotation Pipeline (PGAP) (5). The genome sequence comprised 5,986,764 bp in total, derived from 148 contigs, with an *N*_50_ value of 268,824 bp and a GC content of 35.04%. Strain ZZQ-138 contains 5,901 genes predicted by the NCBI PGAP.

Analysis of pesticidal factors through the whole genome was performed with the online pipeline BtToxin_Digger (https://bcam.hzau.edu.cn/BtToxin_Digger/index.php) (6). Fourteen virulent factors were identified, including one rank 2 (identity of 45% to <76%) and one rank 3 (identity of 76% to <95%) *spp* gene (sphaericolysin-related pesticidal protein) encoding the mosquitocidal toxin sphaericolysin (7), *spp1Aa1* (LAE98_15925 and LAE98_29370, respectively). Those rank 2 and rank 3 *spp* genes may encode novel pesticidal proteins and mechanisms and need further research.

### Data availability.

The draft genome sequence of B. wiedmannii biovar *thuringiensis* ZZQ-138 was deposited in GenBank under the accession number JAIQCN000000000, and the project data are available under BioProject accession number PRJNA760821 and BioSample accession number SAMN21365267. The raw draft genome data were deposited in the Sequence Read Archive (SRA) under SRA accession number SRR15829531.
